# Metrics to evaluate implementation scientists in the USA: what matters most?

**DOI:** 10.1186/s43058-022-00323-0

**Published:** 2022-07-16

**Authors:** Brenna B. Maddox, Mary L. Phan, Y. Vivian Byeon, Courtney Benjamin Wolk, Rebecca E. Stewart, Byron J. Powell, Kelsie H. Okamura, Melanie Pellecchia, Emily M. Becker-Haimes, David A. Asch, Rinad S. Beidas

**Affiliations:** 1 Department of Psychiatry, TEACCH Autism Program, University of North Carolina-Chapel Hill, 100 Renee Lynne Court, Carrboro, NC 27510 USA; 2 Department of Psychology, Utah State University, Logan, UT USA; 3 Department of Psychology, University of California Los Angeles, Los Angeles, CA USA; 4 Department of Psychiatry, Perelman School of Medicine, University of Pennsylvania, Philadelphia, PA USA; 5 Penn Implementation Science Center at the Leonard Davis Institute of Health Economics (PISCE@LDI), University of Pennsylvania, Philadelphia, PA USA; 6 Center for Mental Health Services Research, Brown School, Washington University in St. Louis, St. Louis, MO USA; 7 Center for Dissemination and Implementation, Institute for Public Health, Washington University in St. Louis, St. Louis, MO USA; 8 Division of Infectious Diseases, John T. Milliken Department of Medicine, School of Medicine, Washington University in St. Louis, St. Louis, MO USA; 9 Department of Psychology, Hawai‘i Pacific University, Honolulu, HI USA; 10 The Baker Center for Children and Families, Harvard Medical School, Boston, MA USA; 11 Hawai‘i State Child and Adolescent Mental Health Division, Honolulu, HI USA; 12 Department of Psychology, University of Hawai‘i at Mānoa, Honolulu, HI USA; 13 Department of Medicine, Perelman School of Medicine, University of Pennsylvania, Philadelphia, PA USA; 14 Department of Medical Ethics and Health Policy, Perelman School of Medicine, University of Pennsylvania, Philadelphia, PA USA; 15 Center for Health Incentives and Behavioral Economics, Perelman School of Medicine, University of Pennsylvania, Philadelphia, PA USA; 16 Penn Medicine Nudge Unit, University of Pennsylvania Health System, Philadelphia, PA USA; 17 Department of Medical Social Sciences, Feinberg School of Medicine, Northwestern University, Chicago, IL USA

**Keywords:** Academic journals, Tenure and promotion, Faculty evaluation, Impact, Community partnerships

## Abstract

**Background:**

Implementation science has grown rapidly as a discipline over the past two decades. An examination of how publication patterns and other scholarly activities of implementation scientists are weighted in the tenure and promotion process is needed given the unique and applied focus of the field.

**Methods:**

We surveyed implementation scientists (mostly from the USA) to understand their perspectives on the following matters: (1) factors weighted in tenure and promotion for implementation scientists, (2) how important these factors are for success as an implementation scientist, (3) how impact is defined for implementation scientists, (4) top journals in implementation science, and (5) how these journals are perceived with regard to their prestige. We calculated univariate descriptive statistics for all quantitative data, and we used Wilcoxon signed-rank tests to compare the participants’ ratings of various factors. We analyzed open-ended qualitative responses using content analysis.

**Results:**

One hundred thirty-two implementation scientists completed the survey (response rate = 28.9%). Four factors were rated as more important for tenure and promotion decisions: number of publications, quality of publication outlets, success in obtaining external funding, and record of excellence in teaching. Six factors were rated as more important for overall success as an implementation scientist: presentations at professional meetings, involvement in professional service, impact of the implementation scientist’s scholarship on the local community and/or state, impact of the implementation scientist’s scholarship on the research community, the number and quality of the implementation scientist’s community partnerships, and the implementation scientist’s ability to disseminate their work to non-research audiences. Participants most frequently defined and described impact as changing practice and/or policy. This expert cohort identified *Implementation Science* as the top journal in the field.

**Conclusions:**

Overall, there was a significant mismatch between the factors experts identified as being important to academic success (e.g., tenure and promotion) and the factors needed to be a successful implementation scientist. Findings have important implications for capacity building, although they are largely reflective of the promotion and tenure process in the USA.

**Supplementary Information:**

The online version contains supplementary material available at 10.1186/s43058-022-00323-0.

Contributions to the literature

- This study highlights the importance of examining how implementation scientists are evaluated with regard to tenure and promotion, given the rapid growth of the field and the unique focus of implementation science.
- Implementation science experts identified a significant mismatch between factors weighted as more important for tenure and promotion (e.g., quantity and quality of publications) versus those more important for being a successful implementation scientist (e.g., community partnerships, impact of work on practice or policy).
- Our findings contribute to recognized gaps in the literature for capacity building and advancement of the field.

## Background

As the field of implementation science grows and coalesces, there is a concomitant growing cadre of implementation scientists in academia. Understanding how implementation scientists are evaluated in the tenure and promotion process is important for the long-term viability of the field.

In the USA, decisions about tenure and promotion are typically made based upon the internal and external evaluation of faculty members [1]. In research-focused institutions, faculty typically are judged on the number and size of funded grants and the number and placement of publications [2, 3]. Despite the known challenges with common metrics (e.g., journal impact factors, h-index) [4–7], these are frequently used as guideposts [8, 9]. These traditional metrics may be even more salient when a discipline is less known to reviewers, such as implementation science.

In addition to needing to meet traditional metrics of academia, implementation scientists must also attend to additional activities aligned with tenets of the field, including the use of participatory design [10] and community-academic partnerships [11], the ability to disseminate work to non-research audiences [12], and changes to practice and/or policy [13]. Needing to align with two sets of metrics—one to meet tenure and promotion and one to achieve success in the field of implementation science—may create challenges for implementation scientists. Other fields (e.g., health services researchers, health equity scholars) have encountered similar challenges, including the perception that community-engaged scholarship is not valued in the tenure and promotion review process [14–17].

To address these matters and to provide guidance to the field and tenure and promotion committees, we surveyed implementation science experts to understand their perspectives on how publication patterns and other scholarly activities of implementation scientists are weighted in the tenure and promotion process. We also explored whether these factors are weighted differently for tenure and promotion versus overall success as an implementation scientist. It is important to note that the authors work in the USA and designed a survey that is mostly reflective of the tenure and promotion process in the USA.

## Methods

### Participants

We purposively recruited survey respondents from an international group of implementation science experts. Our list of experts was compiled from (1) individuals listed as *Implementation Science* editors, associate editors, and editorial board members; (2) the AcademyHealth National Institutes of Health (NIH) Annual Conference on the Science of Dissemination and Implementation in Health Committee and Scientific Advisory Board; (3) the NIH Implementation Research Institute core faculty, expert faculty, and fellows; (4) the NIH Mentored Training for Dissemination and Implementation Research in Cancer faculty and fellows; (5) Knowledge Translation Canada experts; (6) the NIH Dissemination and Implementation Research in Health (DIRH) Review Committee; (7) the NIH Training Institute for Dissemination and Implementation Research in Health faculty mentors; (8) the Society for Implementation Research Collaboration (SIRC) Network of Expertise Established Investigators; and (9) the principal investigators of NIH DIRH funded R01s (as of January 2020). The initial recruitment email was sent to 457 potential participants.

### Procedure and measures

The University of Pennsylvania’s Institutional Review Board approved the study procedures. Potential participants received an email from the senior author (RB) inviting them to participate in a brief (i.e., 15–30 min) online survey through REDCap (see Additional file 1 for the full survey). Questions were adapted from previous surveys on faculty evaluation [18]. Specifically, we queried about (1) factors weighted in tenure and promotion for implementation scientists (10 items rated on a 1–3 scale, with higher scores indicating greater influence), (2) how important these factors are for success as an implementation scientist (10 items rated on a 1–3 scale, with higher scores indicating greater importance), (3) how impact is defined for implementation scientists (2 open-ended questions), (4) top journals in implementation science (open-ended question), and (5) how the prestige of these journals is perceived (on a 0–9 scale, with higher scores indicating greater prestige). We also examined the impact factors of the journals with the highest frequencies of implementation science papers. Data collection occurred from April 15, 2020, to May 15, 2020. Individuals received up to three reminder emails, sent weekly after the initial invitation. All participants provided informed consent electronically.

The methods informing the survey section on top journals in implementation science and perceived prestige of these journals were based on a similar study in health services research by Brooks, Walker, and Szorady [19], which involved program chairs rating the level of achievement of faculty who published in specific journals in health care administration. We adapted their survey prompt, replacing “health care administration” with “implementation science.” Participants rated the perceived prestige of 24 journals obtained via bibliometric methods (see Additional file 2 for methods used to generate the list of journals). For all journals reported below, the study team pulled the impact factors from journal websites as of November 1, 2021.

### Data analyses

Quantitative data were analyzed with IBM SPSS Statistics version 28. First, we calculated univariate descriptive and frequency statistics. Next, we compared how participants weighted each of the 10 factors (see Additional file 1) for tenure and promotion versus overall success as an implementation scientist using Wilcoxon signed-rank tests (ordinal, item-level data). Finally, open-ended survey responses were managed in Excel and analyzed by two reviewers independently (BM and MP, or BM and RB) using conventional content analysis involving five steps: reading the data in its entirety, developing codes to reflect the data, coding the data, reviewing the data and codes a second time, and establishing consensus between the coders through discussion [20].

## Results

### Participant characteristics

A total of 132 implementation science experts completed the survey (28.9% response rate). See Table 1 for participant characteristics.

**Table 1:** Participant characteristics (*n* = 132)

	Percentage or mean (*SD*), range
Gender	
Female	50.0%
Male	43.2%
Did not report	6.8%
Age (years)^a^	50.06 (10.84), 27–77
Hispanic/Latinx	5.3%
Race/ethnicity^b^	
American Indian	1.5%
Asian	9.1%
Black or African American	3.0%
White	85.6%
Did not report	3.8%
Highest degree earned^b^	
Master’s degree (e.g., MS, MA)	10.6%
Doctoral degree (e.g., PhD, ScD)	84.8%
Medical degree (e.g., MD, DO)	18.2%
Primary professional role	
Researcher	94.7%
Practitioner	2.3%
Other^c^	3.0%
Country of employment (*n* = 86)^d^	
Australia	2.3%
Canada	8.1%
Denmark	1.2%
Germany	1.2%
New Zealand	1.2%
Sweden	1.2%
UK	3.5%
USA	81.3%
Academic rank	
Assistant professor	17.4%
Associate professor	26.5%
Full professor	47.0%
N/A	9.1%
Number of years working in the implementation science field	13.59 (7.86), 2–45^e^
Ever participated in a committee that makes decisions about tenure and promotion for implementation scientists	46.2%

^a^Nineteen participants did not report their age

^b^Participants could select more than one response

^c^Other professional roles included equal time as practitioner and researcher; administration; teacher, advisor, and mentor; and federal government research staff

^d^Forty-six participants did not share their contact information. For the 86 participants who did share contact information, we determined their country of employment

^e^This response includes participants who considered their experience with implementation science work before the field coalesced under the formal name

### Factors weighted in tenure and promotion decisions

As summarized in Table 2, participants rated the same list of 10 factors for two separate questions to compare the degree of influence for tenure and promotion decisions versus the degree of importance to being a successful implementation scientist. Each of these factors showed significantly different ratings between the two areas. Four factors were rated as more important for tenure and promotion decisions, compared to being a successful implementation scientist: number of publications, quality of publication outlets, success in obtaining external funding, and record of excellence in teaching. Six factors were rated as more important for the overall success as an implementation scientist, compared to tenure and promotion decisions: presentations at professional meetings, involvement in professional service, impact of the implementation scientist’s scholarship on the local community and/or state, impact of the implementation scientist’s scholarship on the research community, the number and quality of the implementation scientist’s community partnerships, and the implementation scientist’s ability to disseminate their work to non-research audiences. Most notably, 65.9% of participants described community partnerships as majorly important to being a successful implementation scientist versus only 12.9% reporting that community partnerships are majorly influential on tenure and promotion decisions.

**Table 2:** Perceived degree of influence/importance of various factors on tenure and promotion decisions for implementation scientists versus the overall success of implementation scientists

Factor	Perceived degree of influence for tenure and promotion decisions about implementation scientists			Perceived degree of importance to being a successful implementation scientist			***Z*** (***p***-value)
	None	Minor	Major	None	Minor	Major	
Number of publications^a^	0%	7.6%	92.4%	2.3%	36.3%	61.4%	− 5.975 (< .001)
Quality of publication outlets^a^	0%	15.9%	84.1%	2.3%	22.7%	75.0%	− 2.468 (.017)
Presentations at professional meetings^b^	12.1%	71.2%	16.7%	1.5%	50.8%	47.7%	− 6.822(< .001)
Success in obtaining external funding^a^	0%	2.3%	97.7%	0%	14.4%	85.6%	− 3.578 (< .001)
Involvement in professional service^b^	3.8%	75.8%	20.4%	6.8%	56.1%	37.1%	− 2.714 (.009)
Record of excellence in teaching^a^	10.6%	65.2%	24.2%	21.2%	65.2%	13.6%	− 3.218 (< .001)
Impact of the implementation scientist’s scholarship on the local community and/or state^b^	12.1%	59.9%	28.0%	3.8%	21.2%	75.0%	− 7.214 (< .001)
Impact of the implementation scientist’s scholarship on the research community^b^	2.3%	29.5%	68.2%	2.3%	15.9%	81.8%	− 2.92 (.005)
The number and quality of the implementation scientist’s community partnerships^b^	25.0%	62.1%	12.9%	2.3%	31.8%	65.9%	− 8.029 (< .001)
The implementation scientist’s ability to disseminate her/his work to non-research audiences^b^	37.1%	56.1%	6.8%	11.4%	46.2%	42.4%	− 7.514 (< .001)

^a^Factor was rated as significantly more important for *tenure and promotion decisions*, compared to being a successful implementation scientist

^b^Factor was rated as significantly more important for being a *successful implementation scientist*, compared to tenure and promotion decisions.

**Fig. 1:**
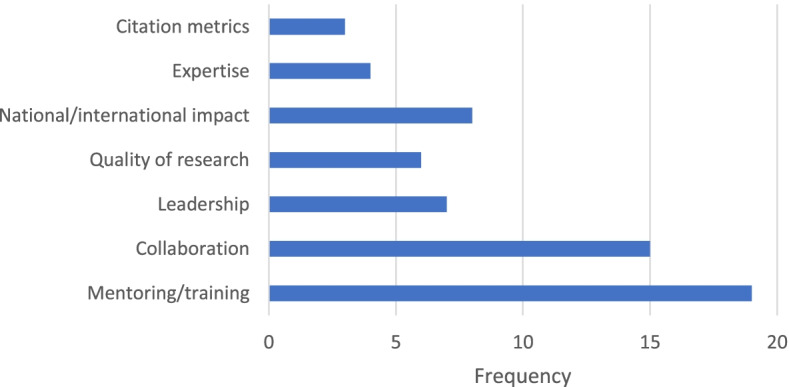
Additional factors reported as important for evaluating implementation scientists on their performance (*n* = 75)

Seventy-five participants shared additional factors perceived as important for evaluating implementation scientists for tenure and promotion. Figure 1 displays the final codes from the content analysis of these open-ended responses. The most frequently described factor was mentoring or training the next generation of implementation scientists. As one participant noted, “Given the state of the field, it is important to have the ability to build capacity in the field through mentorship.” Other factors included collaboration (e.g., ability to conduct team science across disciplines), leadership (e.g., leadership in professional or practice organizations that disseminate evidence), quality of research (e.g., methodological rigor of work), national or international impact (e.g., impact on national policy), expertise (e.g., methodological strength in a specific area), and citation metrics (e.g., h-index).

### Defining and describing impact

**Fig. 2:**
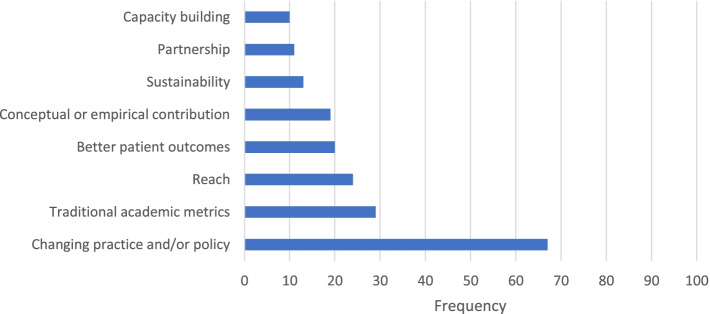
Coded definitions of impact of an implementation scientist’s work (*n* = 106)

**Fig. 3:**
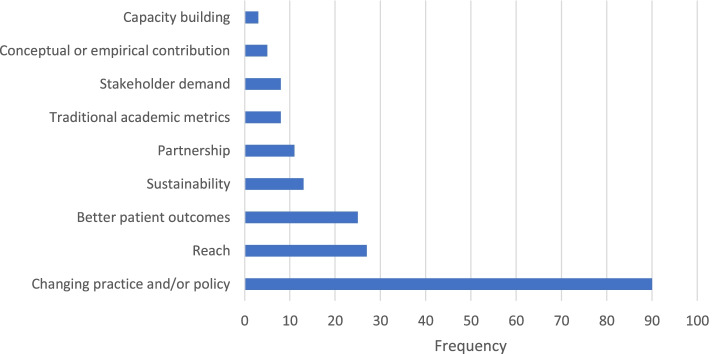
Coded descriptions of participants’ own work having an impact (*n* = 118)

Content analysis of 106 open-ended responses about how best to define impact revealed eight codes (Fig. 2). The same eight codes, plus one additional code, emerged from 118 open-ended responses about a situation when the participant’s work had an impact (Fig. 3). Table 3 displays the definition and an example response for each code. Changing practice and/or policy was the most frequently coded response, reported by the majority of participants for both questions. Of note, six participants expressed uncertainty about their work having an impact, and six participants noted that determining whether work has an impact is difficult because it takes a long period of time. As one participant shared, “You do not know at the time; you may feel your work could have potential, but it takes time to see any impact - this is generally over years.”

**Table 3:** Code definitions and examples from content analysis of impact questions

Code name	Code definition	Code example
Better patient outcomes	Improved patient-level outcomes	“Improvement in the health and well-being of the people we are trying to reach with an evidence-based intervention”
Capacity building	Greater individual, organization, or system capabilities to conduct and implement high-quality research and practice [21–23]	“Student training and mentorship (e.g., developing little D&I-lings)”
Changing practice and/or policy	Practice-wide or policy-level changes	“Policy changed to promote evidence-based practice implementation as a result of implementation work”
Conceptual or empirical contribution	Making a substantial conceptual or empirical contribution to the field	“Helping solve key implementation science methodological and conceptual issues”
Partnership	Collaboration with partners, including community partners and research team collaborators	“Length and depth of connection to local community and state”
Reach	The number of people reached by a policy or intervention and how representative they are of the target population [24]	“How many individuals are touched in the target population”
Stakeholder demand	When community stakeholders (e.g., providers, patients) initiate contact with the implementation scientist to request intervention or expertise	“When community programs kept asking me for my intervention”
Sustainability	Extent to which a program or policy becomes institutionalized or part of the routine organizational practices and policies [24]; maintenance over time	“Project sustained beyond funding from research”
Traditional academic metrics	Traditional metrics for evaluating academic performance (e.g., grants, publications, citations)	“Number of high-profile publications and grants”

### Journal endorsements and ratings

When asked to report the top three journals that publish implementation science papers, almost all participants (97.8%) named *Implementation Science*. The next most frequently named journal was *Administration and Policy in Mental Health and Mental Health Services Research* (20.5%). The journals that were named by ≥ 10 participants as one of the top three are displayed in Table 4 with their impact factors.

**Table 4:** Top journals that publish implementation science (selected by ≥ 10 participants as one of the top three) with impact factors

Journal	Frequency of endorsements	Impact factor
*Implementation Science*	129	7.327
*Administration and Policy in Mental Health and Mental Health Services Research*	27	2.847
*Translational Behavioral Medicine*	25	3.046
*BMC Health Services Research*	21	2.655
*BMJ Quality & Safety*	13	7.035
*Journal of General Internal Medicine*	10	5.128
*Psychiatric Services*	10	3.084
*Implementation Science Communications*	10	N/A
*Implementation Research and Practice*	10	N/A

*Implementation Science Communications* and *Implementation Research and Practice* are newer journals and did not have impact factors available at the time of this study

The participants’ perceived achievement ratings of faculty who published an implementation science paper in each of the journals are displayed in Table 5. *Implementation Science* received the highest achievement rating, which was significantly higher than the second highest rating for the *Journal of General Internal Medicine*, *t*(131) = 7.831, *p* < .001.

**Table 5:** Achievement ratings of faculty members who published an implementation science paper in selected journals (0 = lowest achievement, 9 = highest achievement), with impact factors

Journal	Mean	*SD*	Min	Max	Impact factor
*Implementation Science*	7.80	1.36	4	9	7.327
*Journal of General Internal Medicine*	6.47	1.67	2	9	5.128
*Medical Care*	6.21	1.80	1	9	2.983
*BMC Health Services Research*	5.77	1.67	0	9	2.655
*BMJ Quality & Safety*	5.73	1.85	0	9	7.035
*Journal of the American Medical Informatics Association*	5.67	1.80	0	9	4.497
*Administration and Policy in Mental Health and Mental Health Services Research*	5.55	1.95	0	9	2.847
*Psychiatric Services*	5.34	2.03	0	9	3.084
*BMC Medical Research Methodology*	5.14	1.84	0	9	4.615
*International Journal for Quality in Health Care*	5.05	1.88	0	9	2.038
*Health Care Management Review*	4.97	1.64	0	9	3.328
*AIDS Care*	4.90	1.78	0	9	2.320
*Health Policy and Planning*	4.86	1.54	0	9	3.344
*Journal of Medical Internet Research*	4.85	1.96	0	9	5.43
*Health Research Policy and Systems*	4.84	1.62	1	9	3.318
*The Journal of Behavioral Health Services & Research*	4.65	2.02	0	9	1.505
*International Journal of Medical Informatics*	4.55	1.81	0	9	4.046
*JMIR mHealth and uHealth*	4.43	1.79	0	9	4.77
*Journal of Evaluation in Clinical Practice*	4.30	1.81	0	9	2.431
*International Journal of Integrated Care*	4.29	1.88	0	9	5.120
*Journal of Community Health*	4.23	1.94	0	9	1.883
*Palliative & Supportive Care*	4.23	1.64	0	9	2.257
*Health Promotion International*	4.23	1.76	0	9	2.483
*Supportive Care in Cancer*	4.10	1.81	0	9	3.603

*Implementation Research and Practice*, *Implementation Science Communications*, and *Global Implementation Research and Applications* were not included for achievement ratings because the journal list was based on a systematic review prior to the launch of these new journals

## Discussion

We surveyed primarily US-based implementation scientists to understand how various factors are weighted within the tenure and promotion process for implementation scientists. Our results indicate that traditional academic metrics such as quantity and quality of scholarly publications and external funding are perceived as more influential for tenure and promotion decisions, compared to their importance for being a successful implementation scientist. Although these metrics were still rated as very important for success as an implementation scientist, additional factors were also rated highly, such as community partnerships, impact, and dissemination to non-scientific audiences. These findings suggest that implementation scientists may experience tension in attempting high-quality implementation research, which takes time and effort to accomplish, while also trying to achieve promotion and tenure. This tension has been noted in other fields [14–17]. If academic promotion is meant to reflect success in a field, then standards for promotion need to incorporate these additional metrics [6, 25]. Fortunately, community-engaged scholarship is emerging as a more influential factor in tenure and promotion decisions at some institutions [26–28]. There are also resources available for faculty seeking promotion or tenure based on community-engaged scholarship and for review committee members evaluating community-engaged scholars [29].

In addition to the factors described above, implementation science is fundamentally centered on impact or implementation success. However, the field lacks a commonly used definition for this outcome. Kilbourne et al. [30] define implementation success as “achieving behavioral or clinical improvement in a population when interventions were implemented in multiple settings and scaled up and sustained after the original research on the intervention ended” (p.S783). Similar to our findings, the authors note that impact or success may not be visible for years after the initial implementation study. In addition, work that advances the conceptual and methodological foundation of the field takes time. Overall, determining more proximal metrics of impact and developing a methodology to evaluate implementation success may be worthwhile for implementation scientists in academia.

There are several tools that implementation scientists and evaluating institutions (e.g., universities, funders) can use to systematically assess and report impact. One example is the Translational Science Benefits Model (TSBM) [31], which includes 30 specific and observable indicators of clinical, community, economic, and policy benefits. Another example is the International School on Research Impact Assessment (ISRIA), which is intended to assist organizations in conducting effective research impact assessments for any scientific domain [32]. Structured CV templates that include research translation activities could also address existing inconsistencies in reporting impact [33].

Respondents provided the most frequent and highest endorsement ratings for the journal *Implementation Science*, which is the flagship journal of the field. Our participant sampling strategy targeted editors, editorial board members, and authors of articles in this journal, which may have influenced our results. However, similar findings have been reported elsewhere, with *Implementation Science* leading other rankings of journals for publishing implementation research [34, 35]. A small number of highly regarded journals in the field could limit publication opportunities for implementation scientists. In positive news, there has been a large increase recently in new journals focused on implementation research (e.g., *Implementation Research and Practice*, *Implementation Science Communications*, *Global Implementation Research and Applications*) as well as numerous special issues on implementation science published in discipline-specific journals. This trend likely points to a changing landscape for implementation research with improved visibility and impact.

This study has limitations. First, this study largely reflects academic practice in the USA, and our findings likely do not apply to many other countries with different tenure and promotion processes. Second, our survey relied only on expert input from people who identify as implementation scientists and whose work has earned recognition in the implementation science field. While this ensured our sample had a high familiarity with implementation research, it is possible that rankings of promotion criteria importance would differ in a broader sample, which could include many researchers whose work aligns closely with implementation science, but who use different terminology to describe their work. Third, 47% of respondents were full professors, meaning they have successfully navigated the academic promotion process, and their survey responses may not generalize to implementation scientists with different experiences related to promotion. Fourth, we did not collect detailed information about the participants' work setting, so we do not know if our sample is skewed toward a particular focus (e.g., behavioral health). Survey respondents likely work at institutions with varying criteria and standards for tenure and promotion. Fifth, less than half of the participants reported prior experience serving on a tenure and promotion committee for an implementation scientist. However, the pattern of results remained largely unchanged when excluding participants without this prior experience from analyses (Additional file 3). Sixth, questions in the survey were largely theoretical and asked respondents to reflect broadly on factors of importance; future work might expand on this using candidate vignettes (e.g., sample CVs and scholarly statistics), which may provide more objective assessments of how different candidates are evaluated. Seventh, while our response rate was consistent with prior studies employing similar methodology [33, 34] as well as other online surveys [36], it was overall relatively low; our response rate may have been further hampered by timing, during the start of the COVID-19 pandemic. Eighth, our sample was predominantly White. Ninth, we did not ask respondents about their expertise in other fields that may experience similar challenges (e.g., health equity, community-based participatory research methods). Finally, *Implementation Science Communications* and *Implementation Research and Practice* were endorsed as highly influential, but do not yet have impact factors.

## Conclusions

This study suggests that implementation scientists often experience a tension between what they must achieve for tenure and promotion and what they must achieve to be impactful and successful as implementation scientists. Our findings highlight the need for implementation scientists to adopt a more structured and systematic method for reporting impact and research translation activities more broadly; in turn, academic institutions and funders are called to recognize and credit scholarly activities that impact practice or policy.

## Supplementary Information

**Additional file 1.** Online Survey.

**Additional file 2.** Details about Journals Listed in Survey.

**Additional file 3.** Supplementary Analyses with the Subset of Participants with Experience Participating on Tenure and Promotion Committees.

## Data Availability

The dataset analyzed during the current study is available from the corresponding author on reasonable request.

## Abbreviations

CV: Curriculum vitae
DIRH: Dissemination and Implementation Research in Health
ISRIA: International School on Research Impact Assessment
MT-DIRC: Mentored Training for Dissemination and Implementation Research in Cancer
NIH: National Institutes of Health
SIRC: Society for Implementation Research Collaboration
TIDIRH: Training Institute for Dissemination and Implementation Research in Health
TSBM: Translational Science Benefits Model
