# Supplementary material for: Metrics to evaluate implementation scientists in the USA: what matters most?
Source: Implement Sci Commun. 2022 Jul 16;3:75. doi: 10.1186/s43058-022-00323-0 (PMC9287698; doi:10.1186/s43058-022-00323-0)
Supplement: Supplementary file 1 — Additional file 1. Online Survey. [file 43058_2022_323_MOESM1_ESM.docx]

Additional File 1: Online Survey

**Introduction**

The objective of this study is examine how the publication patterns and other scholarly activities of implementation scientists are weighted in the tenure and promotion process. We plan to email this survey to an international group of implementation science experts. Our list of experts was compiled from advisory committees, editorial boards, training institute mentors, and principal investigators of NIH grants in the field of implementation science.

The first part of the survey is focused on identifying the top journals that publish implementation science related work. Implementation science is a relatively new field, with few journals devoted exclusively to implementation science publications. To date, there has been no systematic effort to rate or rank journals that publish implementation science studies. Our goal with these ratings is to provide some guidance to the appraisal of faculty performance in the field of implementation science.

The second part of the survey is focused on other possible factors in the evaluation of faculty performance. We are interested in whether factors that are associated with being a successful implementation scientist are also weighted heavily in the evaluation for tenure and promotion.

**Journal Ratings**

1. In your opinion, what are the top three journals that publish implementation science papers?

____________________________________________

____________________________________________

____________________________________________

2. How would you rate the achievement of a faculty member who published an implementation science paper in the following journals? Rate their achievement from 0 (lowest achievement) to 9 (highest achievement).

1. Administration and Policy in Mental Health and Mental Health Services Research
2. AIDS Care
3. BMC Health Services Research
4. BMC Medical Research Methodology
5. BMJ Quality & Safety
6. Health Care Management Review
7. Health Policy and Planning
8. Health Promotion International
9. Health Research Policy and Systems
10. Implementation Science
11. International Journal for Quality in Health Care
12. International Journal of Integrated Care
13. International Journal of Medical Informatics
14. JMIR mHealth and uHealth
15. Journal of Community Health
16. Journal of Evaluation in Clinical Practice
17. Journal of General Internal Medicine
18. Journal of Medical Internet Research
19. Journal of the American Medical Informatics Association
20. Medical Care
21. Palliative & Supportive Care
22. Psychiatric Services
23. Supportive Care in Cancer
24. The Journal of Behavioral Health Services & Research

3. Please add the names of any additional journals you believe should be included because they are common outlets for implementation science studies. For each journal added, rate it in the same way you did the journals listed above.

Other: ­­_________________________________ Rating (0-9): __________

Other: ­­_________________________________ Rating (0-9): __________

Other: ­­_________________________________ Rating (0-9): __________

Other: ­­_________________________________ Rating (0-9): __________

Other: ­­_________________________________ Rating (0-9): __________

**Other Questions about Tenure and Promotion Criteria**

1. What degree of inﬂuence do the following issues have on the final decision by faculty committees to recommend or deny tenure and promotion for implementation scientists?

Possible responses: (1) No Influence, (2) Minor Influence, or (3) Major Influence

- Number of publications
- Quality of publication outlets
- Presentations at professional meetings
- Success in obtaining external funding
- Involvement in professional service (e.g., service on committees or editorial boards)
- Record of excellence in teaching
- Impact of the implementation scientist’s scholarship on the local community and/or state (e.g., impact on a clinic, health system, policy)
- Impact of the implementation scientist’s scholarship on the research community (e.g., the scientist produces a widely used framework or measure)
- The number and quality of the implementation scientist’s community partnerships
- The implementation scientist’s ability to disseminate her/his work to non-research audiences (e.g., OpEd piece, blog post)

2. How important are the following factors to being a successful implementation scientist?

Possible responses: (1) No Importance, (2) Minor Importance, or (3) Major Importance

- Number of publications
- Quality of publication outlets
- Presentations at professional meetings
- Success in obtaining external funding
- Involvement in professional service (e.g., service on committees or editorial boards)
- Record of excellence in teaching
- Impact of the implementation scientist’s scholarship on the local community and/or state (e.g., impact on a clinic, health system, policy)
- Impact of the implementation scientist’s scholarship on the research community (e.g., the scientist produces a widely used framework or measure)
- The number and quality of the implementation scientist’s community partnerships
- The implementation scientist’s ability to disseminate her/his work to non-research audiences (e.g., OpEd piece, blog post)

3. Please share any additional factors you think are important for evaluating implementation scientists on their performance (e.g., for tenure, promotion):

____________________________________________________

4. We are interested in the best ways to measure the impact of an implementation scientist’s work. How do you define impact in this context?

____________________________________________________

5. Think about a time when your work had “real-world impact.” What was the situation, and how did you know at that time that your work had impact?

____________________________________________________

**Background Questions**

Please answer the following questions about yourself.

1. Your name (first and last): _________ (include a “prefer not to disclose” option)

2. Your email address: _________ (include a “prefer not to disclose” option)

3. Your year of birth: _________

4. Gender:

____ Female

____ Male

____ Other (please specify: __________)

5. Are you Hispanic/Latino(a)?

____ Yes

____ No

6. Which of the following do you consider your race/ethnicity? (Please check one or more)

____ White/Caucasian

____ Black/African American

____ American Indian

____ Alaska Native

____ Middle Eastern

____ Asian

____ Native Hawaiian or Other Pacific Islander

____ Other (please specify: ______________________)

7. Highest degree earned (more than one can be selected):

____ Bachelor’s degree or less

____ Master’s degree (e.g., MS, MA)

____ Doctoral degree (e.g., PhD, ScD)

____ Nursing degree (e.g., NP, RN)

____ Medical degree (e.g., MD, DO)

____ Other (please specify: ______________________)

8. Primary professional role:

____ Researcher

____ Practitioner

____ Other (please specify: ______________________)

9. Academic rank (if applicable):

____ Assistant professor

____ Associate professor

____ Full professor

____ Other (please specify: ______________________)

____ Not applicable

10. Number of years working in the implementation science field: _________

11. Have you ever participated in a committee that makes decisions about tenure and promotion for implementation scientists?

____ Yes

____ No
