# Supplementary material for: Metrics to evaluate implementation scientists in the USA: what matters most?
Source: Implement Sci Commun. 2022 Jul 16;3:75. doi: 10.1186/s43058-022-00323-0 (PMC9287698; doi:10.1186/s43058-022-00323-0)
Supplement: Supplementary file 2 — Additional file 2. Details about Journals Listed in Survey. [file 43058_2022_323_MOESM2_ESM.docx]

Additional File 2: Details about Journals Listed in Survey

To generate the list of journals that publish implementation science to be included in the survey, we conducted a systematic search of 137 health care sciences, health care services, health policy, and health services journals, based on established categories from the Web of Science Journal Citation Reports. We used PubMed to identify all implementation science articles published in these journals between January 1, 2000 and September 19, 2019. Our method, developed in collaboration with a medical librarian, involved searching *implementation science* in all fields for each of the 137 journals. The search returned 3,300 unique records, which were divided among a team of trained coders to screen for inclusion based on a series of structured questions about whether a study is implementation science, supported by Rayyan software. When the coders were uncertain about whether a study was implementation science or not, they provided a “maybe” rating, and all “maybe” ratings were assigned to co-authors with expertise in implementation science for full text review and final determination. The first author also double coded 10% of all abstracts and held regular meetings with the coding team to discuss any discrepancies, with decisions made by 100% consensus. This process resulted in 1560 abstracts for inclusion, from 72 distinct journals. We included the 24 journals with > 5 implementation science papers in the survey for rating.
