# Supplementary material for: Metrics to evaluate implementation scientists in the USA: what matters most?
Source: Implement Sci Commun. 2022 Jul 16;3:75. doi: 10.1186/s43058-022-00323-0 (PMC9287698; doi:10.1186/s43058-022-00323-0)
Supplement: Supplementary file 3 — Additional file 3. Supplementary Analyses with the Subset of Participants with Experience Participating on Tenure and Promotion Committees. [file 43058_2022_323_MOESM3_ESM.docx]

Additional File 3: Supplementary Analyses with the Subset of Participants with Experience Participating on Tenure and Promotion Committees

The pattern of results remained largely unchanged when we examined only the responses from the 46% of participants who reported having experience participating in committees that make decisions about tenure and promotion for implementation scientists. The exceptions were that this subset of respondents did not give significantly different ratings for quality of publication outlets (*p* = .213), success in obtaining external funding (*p* = .219), and involvement in professional service (*p* = .238) between the two categories of tenure and promotion decisions and being a successful implementation scientist.
